# Morphological, biological, and genomic characterization of a newly isolated lytic phage Sfk20 infecting *Shigella flexneri, Shigella sonnei,* and *Shigella dysenteriae1*

**DOI:** 10.1038/s41598-021-98910-z

**Published:** 2021-09-29

**Authors:** Bani Mallick, Payel Mondal, Moumita Dutta

**Affiliations:** Division of Electron Microscopy, ICMR-National Institute of Cholera and Enteric Diseases, P-33, C.I.T. Road, Scheme XM, Beliaghata, Kolkata, WB 700010 India

**Keywords:** Microbiology, Environmental sciences, Microscopy

## Abstract

Shigellosis, caused by *Shigella* bacterial spp., is one of the leading causes of diarrheal morbidity and mortality. An increasing prevalence of multidrug-resistant Shigella species has revived the importance of bacteriophages as an alternative therapy to antibiotics. In this study, a novel bacteriophage, Sfk20, has been isolated from water bodies of a diarrheal outbreak area in Kolkata (India) with lytic activity against many *Shigella* spp. Phage Sfk20 showed a latent period of 20 min and a large burst size of 123 pfu per infected cell in a one-step growth analysis. Phage-host interaction and lytic activity confirmed by phage attachment, intracellular phage development, and bacterial cell burst using ultrathin sectioning and TEM analysis. The genomic analysis revealed that the double-stranded DNA genome of Sfk20 contains 164,878 bp with 35.62% G + C content and 241 ORFs. Results suggested phage Sfk20 to include as a member of the T4 myoviridae bacteriophage group. Phage Sfk20 has shown anti-biofilm potential against *Shigella* species. The results of this study imply that Sfk20 has good possibilities to be used as a biocontrol agent.

## Introduction

*Shigella* species, one of the most common causes of diarrheal diseases in developing and underdeveloped countries, affects mostly children below 5years^1,2^. Annually 188 million *Shigella* infection causes around 164,000 deaths worldwide^3^. *Shigella* transmitted primarily through the fecal–oral route and a very low number (10–100) of bacteria is sufficient to cause the infection^2^. In developing countries, outbreaks happen mainly due to contaminated food and water, poor hygiene, malnutrition, and lack of awareness^4^. *Shigella* spp. is gram-negative, non-motile, non-spore-forming, rod-shaped bacteria belonging to the family Enterobacteriaceae. There are four different serogroups: *S. flexneri*, *S. sonnei*, *S. boydii* and *S. dysenteriae1* that causes the outbreaks. Among them, *S*. *flexneri* and *S*. *dysenteriae*1 were the causative agents of diarrheal cases predominantly in the developing world^5^. *Shigella* dysentery used to be controlled by antibiotic therapy. Recently, overuse and misuse of antibiotics have increased the number of multidrug-resistant strains of pathogenic bacteria that include *Shigella*^6^. Although there is some progress in vaccine development but an effective vaccine for *Shigella* spp. is yet to achieve^7^. The World Health Organization (WHO) has included *Shigella* in the list of priority pathogens in search of new antibiotics^8^.

Bacteriophages are bacterial viruses that infect specific host bacterial strains without affecting other bacteria. They are abundantly spread in all ecosystems wherever bacteria present^9^ and survive through replicating inside the host cell by controlling its cellular components and releasing mature phage particles by host cell lysis^10^. Phage virulence was found to increase in the presence of human cells than in laboratory bacterial culture^11^. Lytic bacteriophages can infect and kill bacteria within a short period. Therefore, lytic phages are useful biocontrol agents and provide a potential alternative treatment to conventional antibiotic therapy to treat bacterial diseases^12^. The first bacteriophage was isolated at almost the same time but independently by Twort^13^ and Felix d’Herelle^14^. Immediately after the discovery, this phage was used to treat children suffering from severe *S*. *dysenteriae* infection^15^. So far, around 78 lytic Shigella phages have been isolated from environmental samples and showed effectiveness to control shigellosis^16^. But the discovery of antibiotics has pushed the phage treatment in the back foot. Recently certain drug-resistant strains have made treatment of some bacterial infections extremely difficult. In the search for alternative therapy, phage research has regained its importance^17–19^. To qualify as a potential therapeutic candidate, only the killing ability of newly isolated environmental phages is not sufficient^20^. A broad host range, large burst size, and production and storage stability are common characteristics to study. The absence of any toxin or lysogeny gene is also an important determinant to confirm by genome sequencing for therapeutic purposes^21^.

Understanding the phage-host interaction and exploiting the same will gradually increase our understanding to apply them in various medical and biotechnological purposes. Most of the known phages are long-tailed and belongs to the Caudovirales order^22^. The receptor-binding protein present in the phage tail machinery is the main component that initiates the attachment to a specific receptor in the host bacterial cell surface and begins the infection process^23^. Therefore, visualization study of phage-host interaction and a complete lytic cycle of phage provide valuable information. The aim of this study was the morphological, biological, and genomic characterization of a newly isolated lytic *Shigella* bacteriophage to use as a potential biocontrol agent for shigellosis. The interaction of host bacteria and bacteriophage was studied by TEM and SEM to elucidate the lytic cycle.

## Results

### Determination of morphology of phage Sfk20

**Figure 1:**
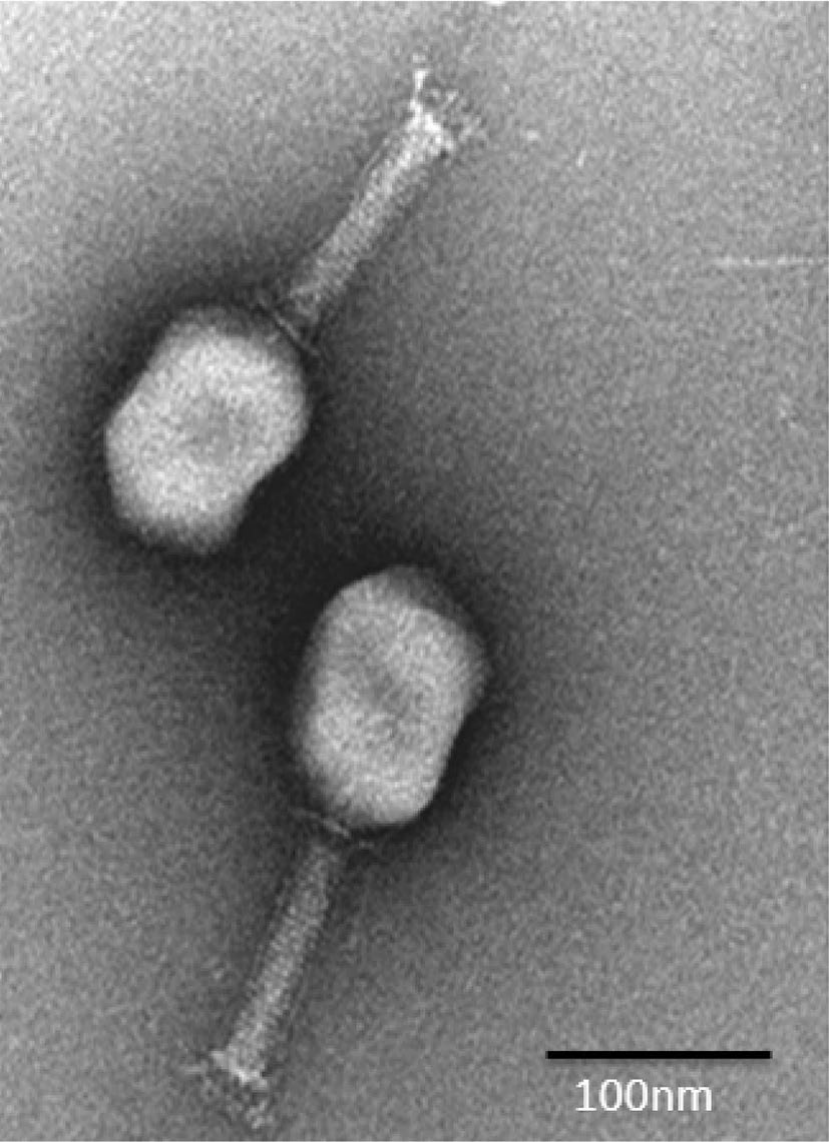
Negatively stained image of bacteriophage particles showing a prolate head, long contractile tail and a baseplate. Scale bar-100 nm.

A lytic bacteriophage, Sfk20 was isolated from environmental water of a diarrheal outbreak area in Kolkata using *Shigella flexneri 2a* 2457T as host strain. TEM analysis revealed that the phage Sfk20 had a prolate head of 91.08 ± 4.92 nm (length), 62.34 ± 4.82 nm (width), n = 20, and a long contractile tail with 99.59 ± 4.92 nm (length) 18.66 ± 2.52 nm width, n = 20 (Fig. 1). Based on the morphology, bacteriophage Sfk20 was classified as a member of the Myoviridae family, Caudovirales order^22^.

### Host range

Host range analysis revealed that Sfk20 could infect *Shigella flexneri* serotypes 1b, 2a, 3*a*, *Shigella sonnei*, *Shigella dysenteriae* 1 but not *Shigella flexneri* serotypes 4,6 and *Shigella boydii* (Table 1). Phage Sfk20 could form clear plaques infecting *Salmonella typhimurium*, *Salmonella enteritidis* but not the *Salmonella typhi strains.* The EOP values obtained for the phage-sensitive *Shigella* strains were much higher compared to the non-typhoidal *Salmonella* strains. However, phage Sfk20 was unable to infect the *E.coli* and *V. cholera* strains used in this study.

**Table 1:** Host range analysis of phage Sfk20.

Bacterial species	Strain No	Infectivity	EOPs
*Shigella flexneri 2a*	2457T	+	1
*Shigella flexneri 3a*	UB811	+	0.80
*Shigella flexneri 1b*	03075/19	+	0.79
*Shigella flexneri 4*	C2529	–	–
*Shigella flexneri 6*	UB812	–	–
*Shigella sonnei*	IDH00968	+	0.62
*Shigella dysenteriae 1*	NT4907	+	0.61
*Shigella boydii 1*	NK02379	–	–
*Salmonella enteritidis*	520833	+	0.000192
*Salmonella typhimurium*	PH-94	+	0.000296
*Salmonella typhi*	KOL 551	–	–
*ETEC*	IDH07942	–	–
*Vibrio cholerae O1*	MAK757	–	–

+  = lysis; – = no lysis.

### Phage stability

**Figure 2:**
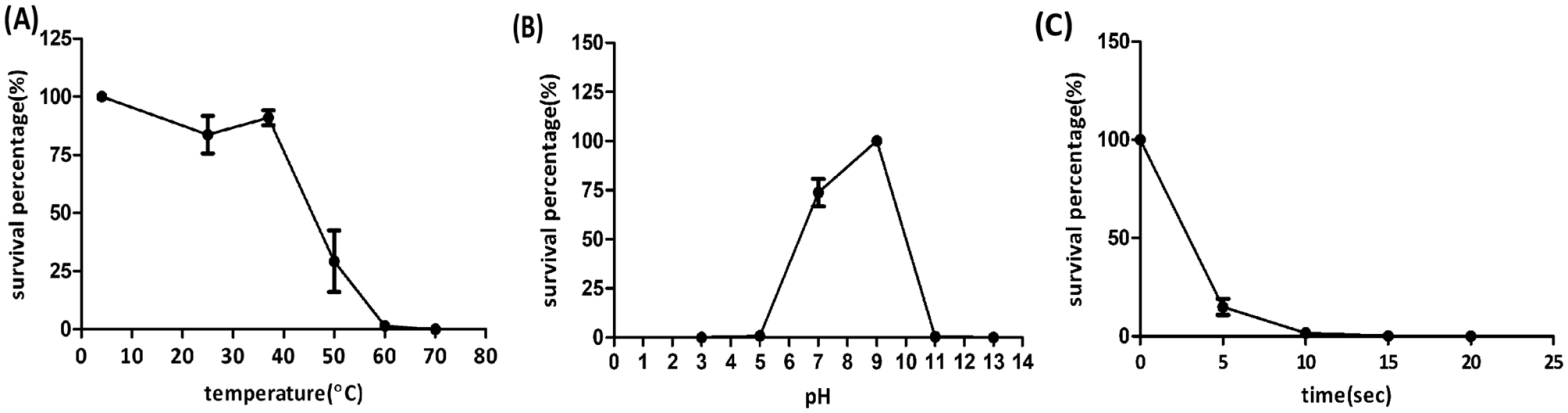
Stability of phage Sfk20: (**A**) Phage thermal stability at different temperature range. (**B**) Phage stability at different pH range, (**C**) Phage stability after UV irradiation.

Phage Sfk20 was tested at wide temperatures and pH values. The thermal stability showed that phage Sfk20 is most stable at 4 °C but decreases rapidly after 37 °C. Phage activity significantly reduced at 50 °C and completely inactivated at 70 °C (Fig. 2A). Phage Sfk20 was found most stable at pH 7–9 range indicating that neutral to slightly alkaline pH is suitable for the activity of phages (Fig. 2B). Phages were completely inactivated below pH 5 and above pH 11. Phage Sfk20 was highly sensitive to UV irradiation and completely inactivated after 15 s (Fig. 2C).

### One-step growth analysis and adsorption rate

**Figure 3:**
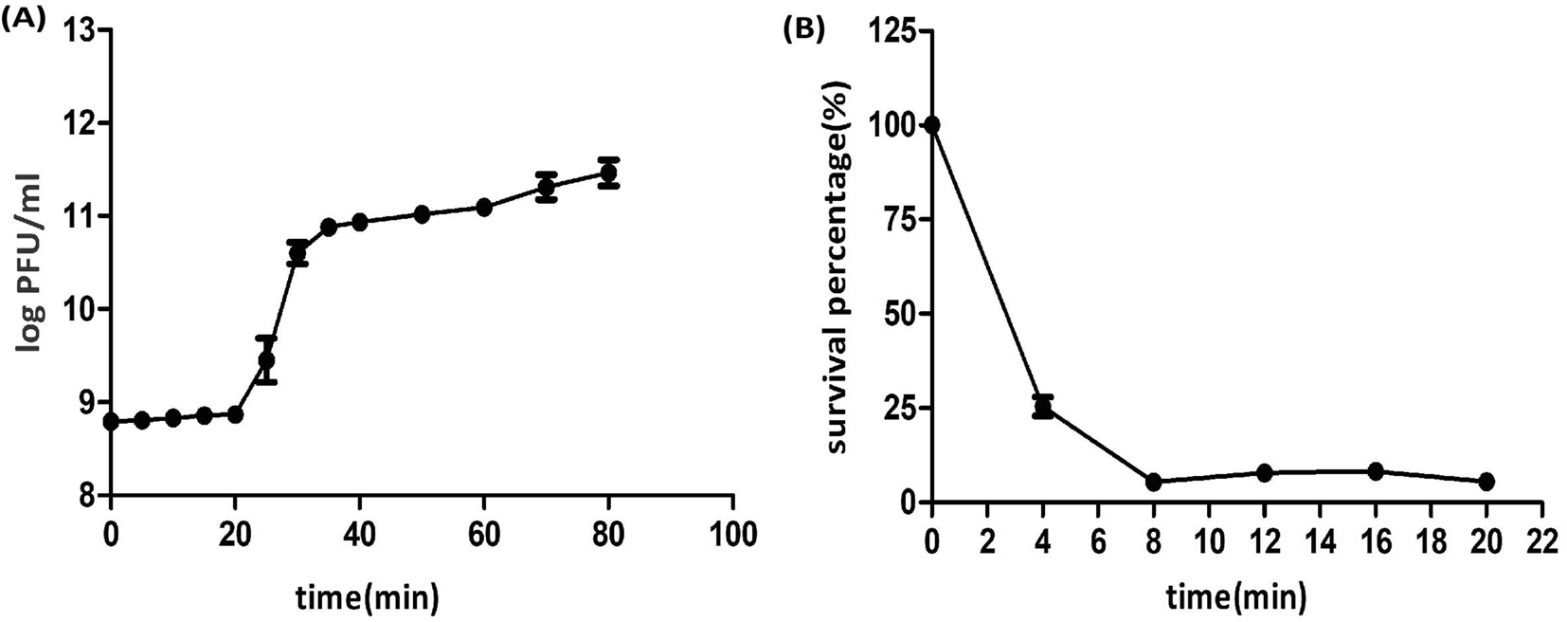
Growth characteristics of phage Sfk20; (**A**) One step growth experiment and (**B**) adsorption kinetics.

The one-step growth curve experiment was performed to calculate the latent period and burst size of the isolated phage Sfk20. As a result, a triphasic curve was obtained with the typical lag phase, burst phase, and stationary phase. A latent period of 20 min and an average burst size of 123 PFU per infected cell was shown in Fig. 3A. Around 94% of bacteriophage particles were adsorbed within the first 8 min. After that, the adsorption rate was very slow shown in Fig. 3B.

### Electron Microscopy of bacteria-bacteriophage interaction

**Figure 4:**
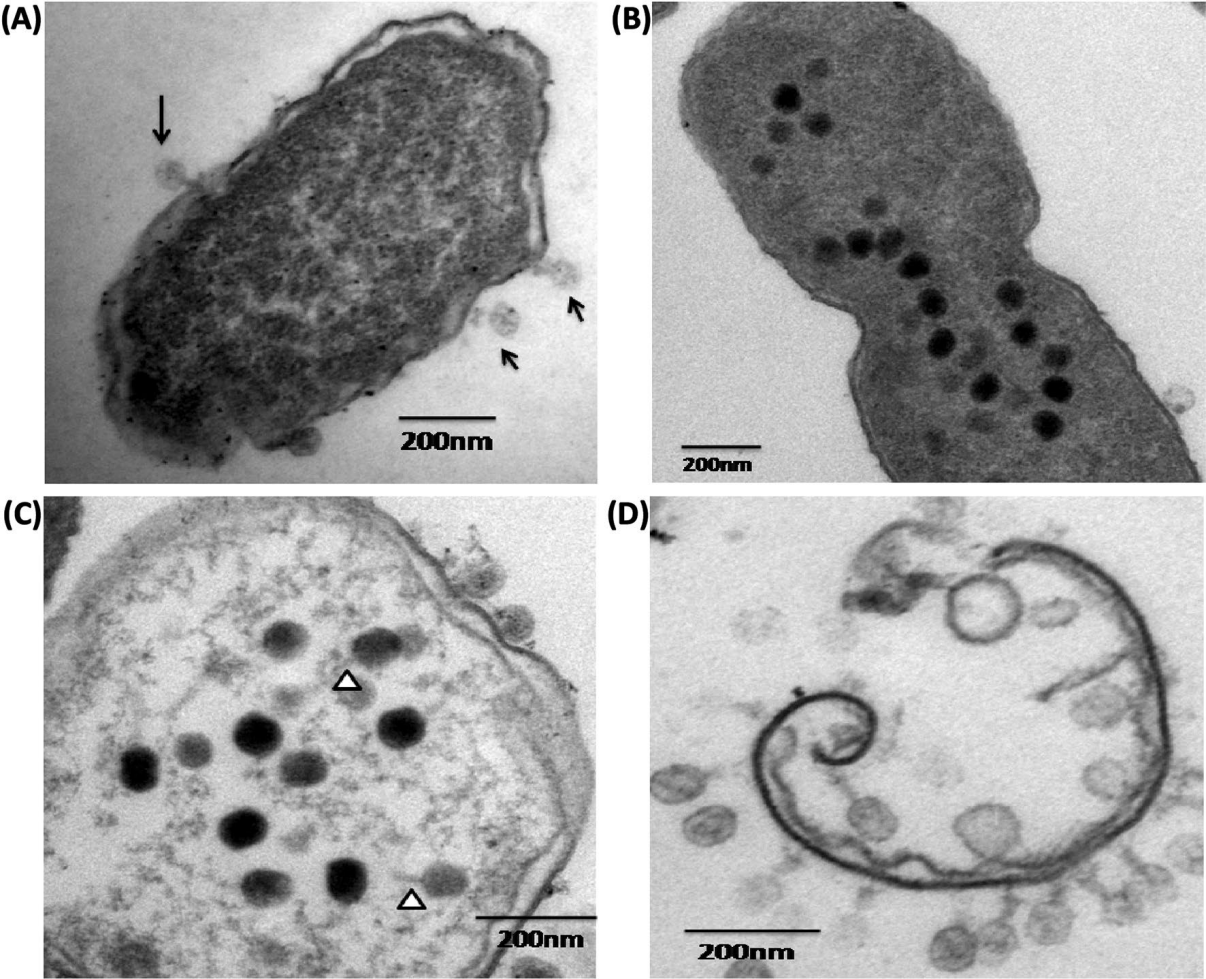
TEM images of ultrathin section of *Shigella flexneri* 2a infected with isolated host specific bacteriophage at different time points: (**A**) Bacteriophage attachment started at 15 min (black arrow indicates phage particles), (**B**) TEM image of 30 min shows intracellular phage development started, (**C**) Intracellular phage, loss of internal material and membrane disorganization is also shown. White arrowheads indicate phage tail. (**D**) Complete lysis of bacteria cell clearly shown at 60 min. Scale bar-200 nm.

Host bacteria *Shigella* *flexneri* *2a* was infected with bacteriophage Sfk20 to visualize the intermediate steps at an ultrastructural level under TEM (Fig. 4). Few bacteriophages are attached to the bacterial cell after 15 min of infection shown in Fig. 4A. The intracellular phage development is visualized at 30 min in Fig. 4B, the loss of internal material and intactness of membrane shown in Fig. 4C. Finally, Fig. 4D showed the complete lysis of the bacterial cell wall around 60 min of infection.

Scanning electron microscopy of bacteria-bacteriophage interaction reveals the different stages of the lytic cycle. Supplementary Fig. S1A shows intact phage Sfk20 particles, followed by the initial step of phage attachment to bacteria (S1B), several phages attached to the bacterial cell surface at a later stage shown in S1C. Finally, disruption of a cell and progeny phages are out around the bacteria are captured in S1D.

### Biofilm degradation study

**Figure 5:**
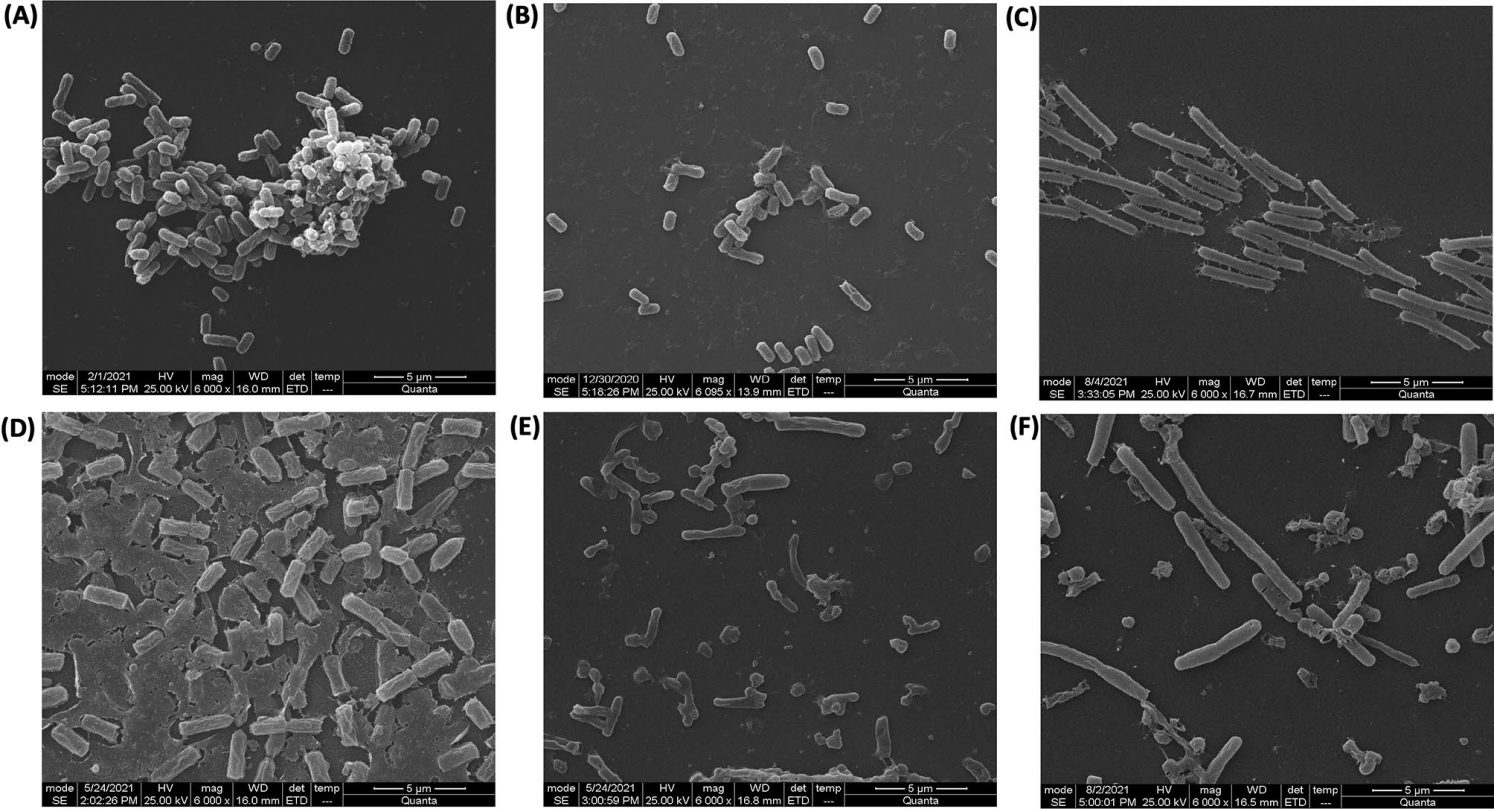
Scanning electron micrograph of biofilm formation and degradation assay. (**A**) 24 h untreated control, (**B**) 24 h biofilm treated with phage Sfk20, (**C**) 24 h biofilm treated with the combination of phage Sfk20 and ampicillin, (**D**) 48 h untreated control, (**E**) 48 h biofilm treated with phage Sfk20, (**F**) 48 h biofilm treated with the combination phage Sfk20 and ampicillin.

*Shigella flexneri 2a* bacterial biofilm grown in a controlled manner on a coverslip was treated with phage Sfk20 alone and a combination of Sfk20 and ampicillin to show its ability to degrade biofilm and results were observed by scanning electron microscopy. Figure 5A and D showed the 24 and 48 h biofilm respectively. The biofilm of 48 h displayed a highly structured matrix formation to which bacteria adhered close to each other compared to 24 h. The phage Sfk20-treated biofilm showed the degradation of the background matrix that results in the more scattered bacterial distribution in Fig. 5B and E. Figure 5C and F showed that the combination of phage Sfk20 and ampicillin reduced the biofilm more significantly and changes were seen in bacterial appearance. The differences between the control biofilm and Sfk20-treated, and Sfk20-ampicillin treated biofilm showed the efficacy of phage Sfk20 in the application of biofilm removal. The Sfk20-ampicillin combination exerted better removal activity than Sfk20 alone.

A quantitative assay of biofilm degradation activity revealed that phage Sfk20 (10^10^pfu/ml) and ampicillin (256 µg/ml) alone caused ~ 40% (*p* < 0.001) and ~ 31% (*p* < 0.001) reduction in biofilm respectively when compared to the control (without phage). However, the effect of the Sfk20-ampicillin combination showed a higher reduction (~ 47%, *p* < 0.01) in biofilm than that of Sfk20 and ampicillin alone. Also, each pair of combinations showed a statistically significant reduction in biofilm (Supplementary Fig. S2). Therefore, a considerable synergistic effect was observed when biofilm was subjected to phage Sfk20 and ampicillin combination.

### Protein profile

The structural protein profile of the bacteriophage Sfk20 were analyzed by 12.5% SDS-PAGE (Supplementary Fig. S3). The two major bands appeared at 53 kDa and 70 kDa. The other lighter bands appeared approximately at 13, 76, 100, and 155 kDa respectively.

### Restriction enzyme digestion of the phage genomic DNA

The restriction digestion pattern is a quick and cost-effective method to detect differences among new phages. Supplementary Fig. S4 showed the pattern of restriction digestion of the phage Sfk20 DNA. Based on this result, the genome of phage Sfk20 carries double-stranded DNA. The phage DNA was cut by EcoRV only but resistant to EcoRI, BamHI, HindIII, PstI, BglII, and MluI restriction enzymes. *Shigella flexneri* phage vB_SflS-ISF001 was also reported resistant to EcoRI and BamHI but sensitive to EcoRV^24^. *Shigella* phages vB_SflM_004 and vB_SsoS_008 were also found digested with only EcoRV^25^.

### Genome sequencing and comparative genomic analysis of Sfk20

**Figure 6:**
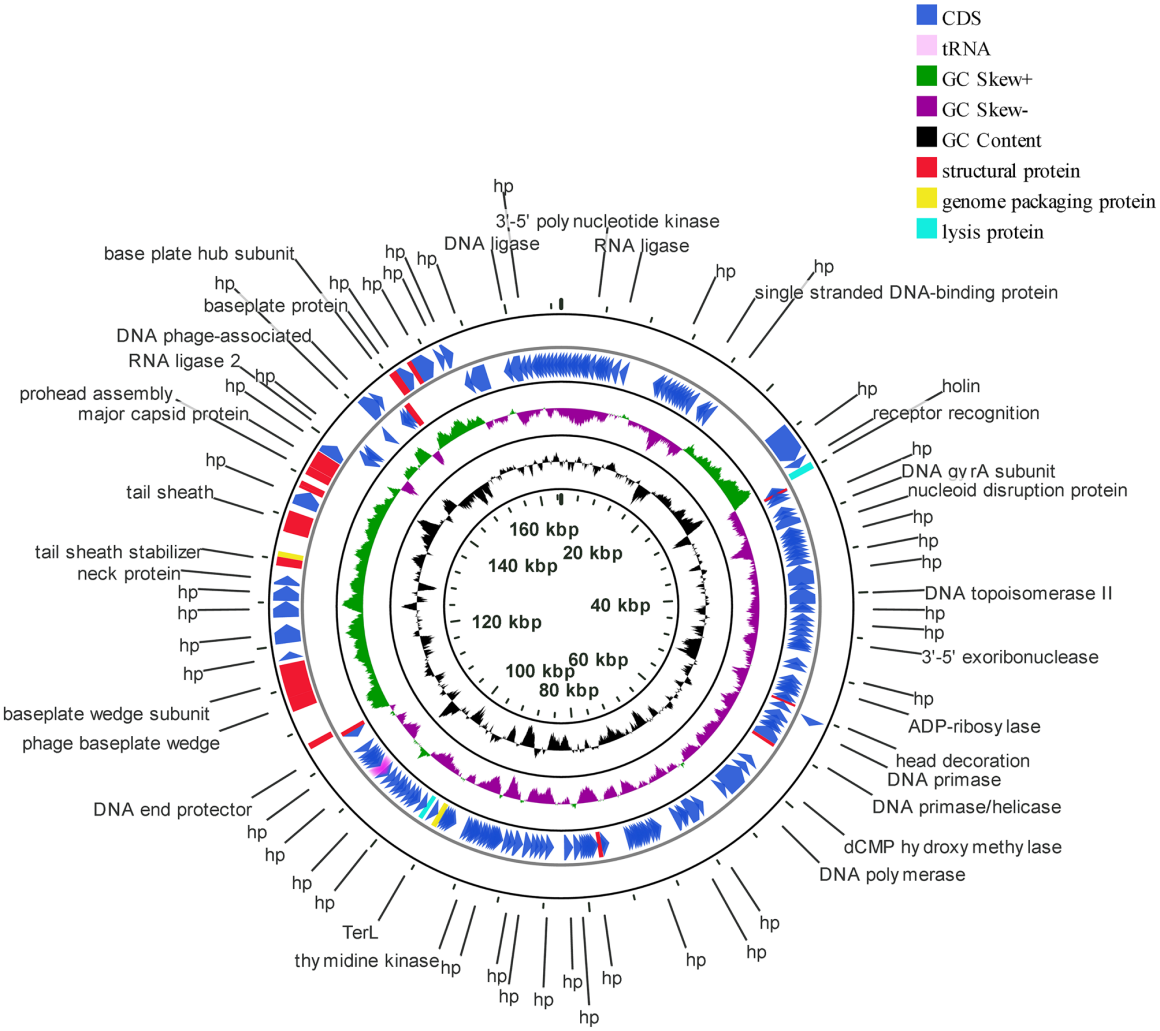
Schematic map of phage Sfk20 genome prepared using CGView. Outer ring represents coding sequence locations (CDS) in blue color; tRNA was marked in pink color. Hypothetical proteins were denoted as hp. The different functional ORFs were indicated in different colors. Red ORFs represent the structural proteins, yellow ORFs represent the genome packaging proteins and the cyan ORFs represent lysis proteins.

**Figure 7:**
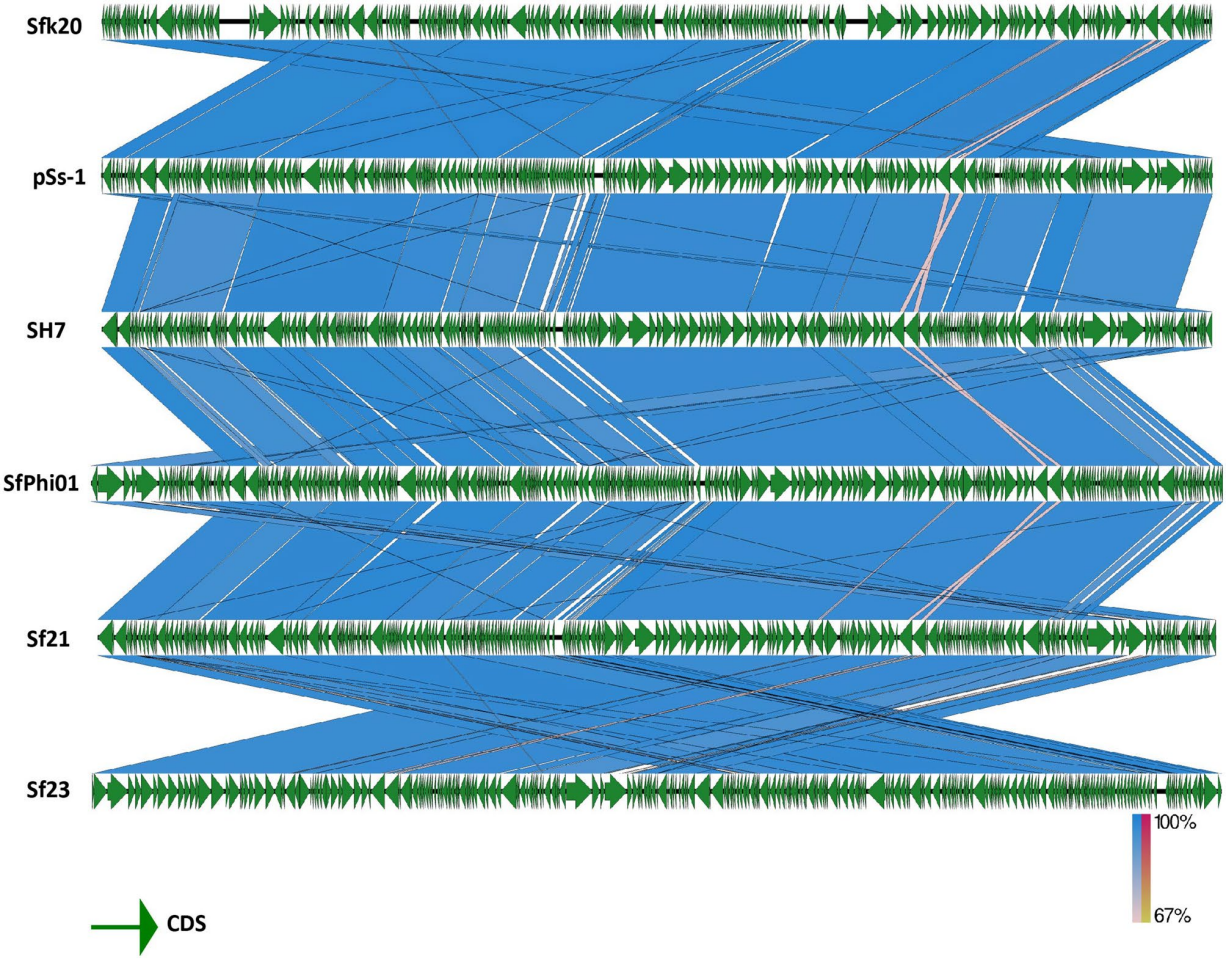
Easyfig schematic alignment of phage Sfk20 genome with five closely related phages using BLASTn program. Green arrows indicate the coding sequence location shaded blue lines indicate degree of homology between pairs.

Phage Sfk20 genome has been sequenced and deposited in GenBank with accession number: MW341595. Genome analysis showed that the phage Sfk20 genome consists of 164,878 bp with a 35.62% total G + C content. Figure 6 represents the schematic genome map of phage Sfk20 drawn using the CG view server. The inner ring represents coding sequence locations (CDS) colored in blue; tRNA in this ring is marked in pink color. The ORFs were mainly annotated as structural proteins, genome packaging proteins, and lysis proteins. Further analysis identified 241 open reading frames (ORFs) and the predominant start codon was ATG (97%). GTG is an uncommon start codon for six ORF such as ORF19, ORF68, ORF76, ORF89, ORF104, and ORF118. Concrete gene information such as the positions, directions, amino acid range, putative function of each ORFs, and their function in phage life cycle was mentioned in Supplementary Table S1. The longest ORF of phage Sfk20 was ORF36 (protein-id: QPP47031) which was placed in the morphogenesis cluster. ORF36 encoded a gene that was very similar to the large distal long tail fiber subunit of Myoviridae Shigella phage SH7. ORF166 (protein_id: QPP47161) represents the phage-encoded enzyme lysozyme/endolysin and ORF38 (protein_id: QPP47033) encodes the protein holin responsible for bacterial lysis. These genes are crucial for host cell destruction during the burst phase of the phage life cycle. They are also called host lysis proteins. The DNA packaging genes such as large terminase subunit, small terminase protein, and putative terminase subunit located in ORF163, ORF198, and ORF199 respectively. Phage morphogenesis genes such as head completion protein, baseplate wedge subunit, neck protein, tail sheath stabilizer, and completion protein, prohead core protein, capsid and scaffold protein, prohead assembly protein, major capsid protein and baseplate hub subunit are located in ORF186, ORF189, ORF195, ORF197, ORF203, ORF204, ORF206, ORF207, and ORF219 respectively. Most of the packaging and morphogenesis genes are located in the middle and end of the genome. In addition, proteins responsible for phage nucleotide metabolism are DNA gyrase subunit, DNA endonuclease IV, DNA topoisomerase II, DNA primase, DNA polymerase, and endonuclease located in ORF46, ORF53, ORF59, ORF84, ORF96, and ORF122 respectively. The presence of nucleotide metabolism-associated genes in this phage indicates that the Sfk20 genome might reduce the dependence of phage DNA metabolism on the host bacteria. A total of 10 tRNA genes were identified in phage Sfk20. The functions of tRNA in the phage genome are still not clear though a widely accepted fact is phage tRNA gives considerable independence from host translational machinery^26^. A Megablast search of phage Sfk20 genome indicated it has a 95–97% sequence similarity to *Shigella* phage pSs-1, SH7, SfPhi01, Sf21, Sf23 (Supplementary Table S2). According to Megablast results, Sfk20 was classified as a member of the T4-like virus genus and myoviridae family. The alignment of phage Sfk20 genome with closely related five other myoviridae *Shigella* phages was represented by Easyfig (Fig. 7). The green arrows represent the coding sequence locations and blue shaded lines reflect the degree of homology between Sfk20 and other phages.

### Phylogenetic position of phage Sfk20

Based on the base plate wedge protein sequences and terminase large subunits (TerL), the phylogenetic trees were generated to find the phylogenetic position of phage Sfk20 in this study (Supplementary Fig. S5). From the NCBI database, the reference sequences were collected. According to Phylogeny.fr dendrogram (http://www.phylogeny.fr/), phage Sfk20 belongs to T4-like virus genus, myoviridae family and caudovirales order.

## Discussion

The rapid increase of multidrug-resistant *Shigella* strains has become a global burden in diarrheal diseases. Virulent lytic phages can selectively infect and destroy the bacterial population including drug-resistant strains, using a mechanism distinct from antibiotics. Therefore, there is a renewed interest to control the MDR *Shigella* strains using phages as an alternative to antibiotic treatment. The Myoviridae bacteriophage Sfk20 used in this study appeared as a promising biocontrol candidate against shigellosis.

Studies have been reported to show the in vitro antimicrobial activity of many isolated phages and successful application of phage therapy since the early nineteenth century in a staggered manner. Many promising laboratory studies have failed to show positive outcomes in the recent controlled human trial^27^. Early phage therapy mostly failed due to inadequate knowledge of the fundamental phage biology and unsophisticated purification and storage procedure. Phages have beneficial characteristics but with some restrictions. Unknown gene function of phages, CRISPR-cas, phage stability, host immune responses, and scarcity of pharmacokinetic and pharmacodynamics models for dose optimization are some genuine concerns to implement phage therapy as an approved alternative. Also, the low pH sensitivity, enzymatic degradation, phage loss, and phage-neutralizing antibodies are some of the big concerns in human phage therapy. Meanwhile, compassionate phage therapy is in limited use in cases when all available therapeutic options are exhausted^28^.

In this study, the water sample was collected from a diarrheal outbreak area and *Shigella flexneri 2a* bacteria was the host for phage propagation. Host range analysis and high EOP values showed *Shigella flexneri* serotypes 1b, 2a, 3a are sensitive towards phage Sfk20 whereas *Shigella flexneri* serotypes 4 and 6 are resistant towards phage Sfk20. In developing countries, the predominant serotypes of *Shigella flexneri* are 1b, 2a, 3a, 4a, and 6 though serotype 2a is predominant in industrialized countries including India^29^. *Shigella flexneri* has been reported to progressively developing antibiotic resistance^30^. So we have chosen this as the host strain in our study. *Shigella boydii* was rarely reported worldwide compared to other *Shigella spp*^31,32^ and there are limited studies published on *S. boydii*^33^. The Global Enteric Multicenter Study (GEMS) reported that only 5.4% of 1130 *Shigella* isolates were identified as *S. boydii*^34^. One study from India reported *S. flexneri* (60%) as the prevalent serogroup followed by *S. sonnei* (23.8%), *S. dysenteriae* (9.8%), and *S. boydii* (5.7%)^35^. *S. boydii* has been reported one of the prevalent serotypes only in Bangladesh^36^.

The stability of phages at varying temperatures and pH can be considered to make them suitable candidates for therapeutic purposes. Phage Sfk20 was found quite stable in the range 4–37 °C and for at least up to six months without losing viability in the usual storage temperature of 4 °C. It showed maximum stability in the range of pH 7–9 that is neutral to slightly alkaline. This information is valuable to consider the administration of phages through the oral route for therapeutic purposes. The acidic environment of the gastrointestinal tract is one of the major challenges for phage therapy during phage administration^37^. Most of the bacteriophages are found sensitive towards low pH. Phage Sfk20 also showed complete inactivity at pH < 5. Therefore, various methods and technologies have been developed to protect the therapeutic phages. Oral applications of phages predated by gastric neutralization are a practice in few European countries where phage therapy has always received substantial consideration^38^. Also, encapsulation of phages in liposomes or biopolymeric microparticles is another highly accepted technology to enhance phage survival while passing through the gut^39^. However, the selection of biopolymer is not so easy and the synthesis process has to satisfy certain features. To overcome this limitation, an alternative has been developed based on genetic engineering by displaying phospholipids on the surface of the phages. This natural coating process not only protects from the acidic pH of the GI tract but also maintains the infection ability of phages^40^. Previously the genetic engineering was quite difficult but with the development of a new technique Bacteriophage Recombineering of Electroporated DNA (BRED)^41^, the coating has become easy and cheaper than encapsulation. Therefore, the sensitivity of Sfk20 at low pH could be controlled during its trial in future phage therapy.

The one-step growth curve of phage Sfk20 suggests a latent period (20 min) and a large burst size of 123 pfu per cell. A large burst size favours this phage's application in phage therapy. Earlier reports on T4 revealed that if tRNA genes were deleted the burst size and protein synthesis rate eventually lowered down^42^. But some reports revealed that bacterial physiological states could affect the burst size of phages^43^.

In this study, we have used early log phase bacterial culture of *S. flexneri 2a* strain. Understanding the interaction of the bacteria-bacteriophage system is an approach to consider bacteriophages as biocontrol agents. Ultrastructural analysis of phage-infected bacteria and intracellular development of phages by transmission and scanning electron microscopic images interpret the morphogenetic pathway of phage Sfk20 and further showed similarities with T4 phage^44^. Further, the different forms of the infected cells in Figs. 4B and C indicated that the extent of intracellular phage development depends on the multiplicity of infection of an individual particle.

Biofilm degradation activity of phage Sfk20 alone and in combination with ampicillin was analyzed by scanning electron microscopy (SEM) (Fig. 5). Penetration, diffusion, and propagation are three important criteria to use single phages or phage cocktails to remove biofilms^45^. The results show phage Sfk20 could fulfil the criteria to a significant extent by degrading the biofilm of its host indicator bacteria *Shigella flexneri 2a*. Phage-ampicillin combination showed a synergistic effect (Fig. 5E) in the removal of *Shigella* biofilm though not complete removal. To the best of our knowledge, Sfk20 is one of the first lytic, tailed *Shigella* phages capable of degrading the biofilm formed by *Shigella flexneri 2a* strain in laboratory conditions. The bacterial biofilm has an extracellular matrix that acts as a physical barrier to the phage. Tailed phages can invade the matrix layer by the hydrolytic activity of depolymerase enzymes present in their tail spike proteins^46^. After that probably phages diffuse through the biofilm to infect and destroy bacteria in the deeper layer^47^. Sfk20 with its long contractile tail might help to remove the external matrix by its enzymatic activity. The other possible reasons for Sfk20 efficacy in biofilm removal are the presence of endolysins and virion-associated peptidoglycan hydrolases (VAPGHs) that either disrupt or make a small hole in the cell^48,49^. Moreover, a small percentage of stress-tolerant cells are usually present in biofilm and a recent study suggests phage lytic proteins can remove that cells^50^. It could probably facilitate the dispersion of the biofilm matrix. This study indicated structural characteristics and spatial information of the bacterial biofilm and qualitative observation of the disruption of the biofilm through direct imaging. The biofilm degradation capacity of Sfk20 revealed that it could be used in the future as a single phage or in a phage cocktail or phage-antibiotic combination as a therapeutic candidate for biofilm-mediated diseases.

We have also done a quantitative experiment to address the eradication efficacy. The use of bacteriophage Sfk20 (10^10^pfu/ml) with ampicillin at a high concentration (256 µg/ml) exhibited a better biofilm removal activity compared to the phage or antibiotic alone (Supplementary Fig. S2). This could be due to the presence and enzymatic activity of depolymerase in the phage tail. It might have degraded the extracellular capsular polysaccharide and phages could diffuse easily inside the biofilm to target bacteria in the deeper layer creating a hassle-free path for a higher dose of antibiotics to reach inside the biofilm. Thus a combination of phage plus antibiotic showed faster destruction of biofilm matrix and the associated bacteria. Similar results were observed in other phage-antibiotic combination therapy^51,52^. Phage-antibiotic synergism is observed in most of the infections caused by biofilm-producing bacteria^53^. Phage Sfk20 alone can remove at least 40% of the total biofilm and in combination with ampicillin, the removal percentage of biofilm was 47%. It confirmed the synergistic effect of the Sfk20-ampicillin combination.

Genome sequencing, analysis, and complete annotation provide sufficient information on the phage Sfk20 genome. Analysis of phage Sfk20 genome confirmed the presence of lytic proteins and absence of any lysogenic, toxin, or antibiotic resistance genes suggesting that the phage could be used as a safe biocontrol agent for host enteric bacteria. Holin and endolysin genes encode proteins responsible for progeny phage release at the end of a lytic cycle. The presence of both genes in the sequence of phage Sfk20 genome can confirm the lytic nature of this phage.

According to the mega blast search result, Sfk20 belongs to the genus T4-like viruses; order caudovirales; family myoviridae; subfamily Tevenvirinae. Recently a total of 78 isolated *Shigella* phages were grouped according to family and genome size and phage Sfk20 falls in the largest T4 like myoviridae family with a genome size 164.0–176.0 kbp^16^. Comparative genome analysis and biological properties suggested that phage Sfk20 has the highest similarity with pSs-1 [Supplementary Table S3].

Phylogenetic tree analysis of Sfk20 based on base plate wedge subunit and terminate large subunit with other related phages suggested that Sfk20 was closely related to other *Shigella* and *E. coli* phages and they probably evolved from a common ancestor.

In conclusion, a constant search for novel lytic bacteriophages during a seasonal upsurge of a diarrheal outbreak will indicate the presence of related virulent bacteria in the environment. Characterization of those phages will facilitate understanding phage biology and the strategy development for effective therapeutic applications.

## Materials and methods

### Isolation, purification and enrichment of bacteriophage

*Shigella flexneri 2a*-specific bacteriophage was isolated from the water sample of a diarrheal outbreak area in Kolkata. A mixture of 25 ml water sample, 25 ml 10× phage broth media and 5 ml of log phase *Shigella flexneri 2a* culture was incubated at 37 °C for 24 h in shaking condition. After this incubation the suspension was centrifuged for 10 min at 10,000 rpm to remove the bacterial cells and the collected supernatant was filtered with a 0.22 µm membrane filter. Spot assay was performed initially to evaluate the presence of shigella phage testing on *Shigella flexneri 2a* 2457T using the double layer agar method. The single plaque isolation procedure was performed thrice by plaque assay to obtain the purified phage plaque. The phage suspension was concentrated and purified by ultracentrifugation (25,000 rpm, 1:30 h and 4 °C) and sucrose step-gradient ultracentrifugation (30,000 rpm, 2 h and 4 °C) respectively^54^. The purified high titre phage Sfk20 phage stock was stored at 4 °C for further studies.

### Transmission electron microscopy

Purified high titre bacteriophage was negatively stained with 2% (w/v) uranyl acetate. The sample was examined under FEI Tecnai 12 BioTwin transmission electron microscope at an operating voltage 100 kV. Detailed morphology that includes the shape and size of bacteriophage particles are studied.

### Host range determination and efficiency of plating (EOP)

The infectivity of Sfk20 was tested against many bacterial strains as determined by standard spot assay^55^. Infectivity was tested on different enteric bacteria present in the laboratory. Briefly, 10 µl of phage (1.8 × 10^11^pfu/ml) sample was spotted onto the middle of bacterial lawn and the plate was incubated overnight at 37 °C and checked for the presence visible lysis zone. The EOP values are calculated as the ratio of the PFU value of phage with susceptible bacterial strain and the phage with indicator (*Shigella flexneri* 2a, 2457 T) bacterial strain.

### Phage stability assay

To evaluate the heat resistant capacity of phage Sfk20, thermal stability was performed. The phage suspension (diluted in Tris MgCl_2_ buffer) was incubated at different temperatures (4 °C, 25 °C, 37 °C, 50 °C, 60 °C, and 70 °C respectively) for 1 h and phage suspension was titred by soft agar over layer method^56^.For pH stability test, phage Sfk20 was examined by preincubating the phage suspensions ranging from pH 3–13 at 37 °C for 1 h. After the incubation period, the phage titre was determined by double agar layer method. To evaluate the phage stability under UV light, the phage suspension was kept at a different time interval of 5, 10, 15, and 20 s adapted and modified from elsewhere^54^. After that, the survival percentage of bacteriophage tested by the soft agar overlay method. All experiments were performed in triplicate.

### One-step growth analysis of bacteriophage and adsorption kinetics

An early exponential phase culture (20 ml) of *Shigella flexneri 2a* 2457T (OD_600_ = 0.5) was harvested by centrifugation (5000×g at 4 °C for 10 min). The pellet was resuspended in around 1 ml Luria broth followed by the addition of bacteriophage at an MOI 0.1. The mixture was incubated for adsorption at 37 °C for 5 min and diluted in Luria broth with a maximum volume of 10 ml and reincubated at 37 °C. Aliquots were taken out at different time intervals up to 80 min to calculate the phage titer by the soft agar layer method.

### Bacteriophage infection and intracellular development of phage

*Shigella flexneri 2a* bacterial culture was mixed with Sfk20 bacteriophage and incubated at 37 °C. Samples were taken out at around 15, 30, and 60 min respectively and were immediately centrifuged at 7000 rpm for 5 min. In the next step, cell pellets resuspended in 3% glutaraldehyde in 0.1 M sodium cacodylate buffer to fix the cells^57^.

### Ultrathin sectioning of the bacterial cell

In this experiment, cacodylate buffered glutaraldehyde was used as a primary fixative. After that, secondary fixation was done in 1% OsO_4_ followed by dehydration with a series of ascending concentrations of ethanol. Then the samples were embedded in resin Agar100 and polymerization was done overnight at 60 °C. The ultrathin sections (40–50 nm) of the control and infected cells are cut with a Leica Ultracut UCT ultramicrotome (Leica Microsystems, Germany). The sections were picked up in nickel or copper grids, dual-stained with 2% aqueous uranyl acetate and 0.2% lead citrate, and grids were air-dried. The grids with sections were either stored or immediately examined under an FEI Tecnai 12 Biotwin TEM (FEI, Netherlands) at an accelerating voltage of 100 kV.

### Scanning electron microscopy

For SEM analyses, to visualize the bacteriophage lytic cycle, samples were taken out from bacteria-bacteriophage mixture at early, mid, and late phase in Eppendorf tubes. Then samples were fixed with 3% glutaraldehyde in 0.1 M sodium cacodylate buffer and left overnight. After that, samples were dehydrated with ascending concentration of alcohol up to 100%. All dehydrated samples were treated with hexamethyldisilazane (HMDS) for chemical drying by increasing HMDS concentration stepwise up to 100% HMDS. The tubes were left in a fume hood overnight to evaporate HMDS completely. Samples mounted on specimen stubs, sputter-coated with gold, and images captured on an FEI Quanta 200 scanning electron microscope (FEI, Netherlands).

### Biofilm degradation assay and visualization with SEM

A qualitative biofilm degradation activity of Sfk20 on *Shigella flexneri 2a* 2457T strain was determined following a previous method with some modification^58^. Cover slips were used for the formation of biofilm. Briefly 10 µl of overnight culture of *Shigella flexneri 2a *was incubated on coverslips submerged in LB media in 6-well plates and incubated at 37 °C for 24 h and 48 h respectively. The biofilm was then treated with phage Sfk20 (10^10^PFU/ml), ampicillin (256 µg/ml) and a combination of both at same individual concentration. All treated samples were further incubated at 37 °C for overnight. After that, planktonic cells were removed and the coverslips were processed for SEM analysis as mentioned in visualization of bacteria-phage interaction method.

### Quantitative assay of biofilm degradation

The quantitative experiment on the ability of Sfk20 to degrade the biofilm alone or in combination with antibiotic was also performed. Briefly, matured biofilm was grown on microtitre plate^58^, planktonic cells were removed and the biofilm was treated with phage Sfk20, ampicillin and their combination^59^. After the treatment the plates were incubated at 37 °C for overnight. Next day the phage and antibiotic were removed and the wells were rinsed with 1× PBS twice and stained with crystal violet. The total biomass of the biofilm was measured by plate reader at the absorbance of 595 nm (iMark Microplate Reader S/N 21673). Statistical analysis of the data was performed by two- way ANOVA with Graph-Pad Prism 5.00.

### SDS-PAGE analysis

Phage suspension was boiled for 5 min and the structural proteins were extracted. The denatured proteins were separated by 12.5% SDS–polyacrylamide gel as described by Laemmli^60^ with Mini-PROTEAN TGX Precast gels (Biorad, USA).

### Phage DNA extraction and restriction endonuclease digestion

Bacteriophage DNA was isolated from the purified phage suspension using the DNA isolation kit (NorgenBiotek Corp., Canada) according to the instruction from the manufacturer. Phage genomic DNA was digested with the restriction endonuclease enzymes (EcoRI, BamHI, HindIII, PstI, EcoRV, BglII, and MluI) according to the supplier’s protocols. DNA fragments were separated by electrophoresis at 100 V for 1 h on a 1% agarose gel and stained with ethidium bromide. DNA molecular marker (high range DNA ladder, HiMedia) ranging from 250 bp to 25 kbp was used.

### Genome sequencing of phage Sfk20

Phage genome DNA was sequenced by Xcelris (Ahmedabad, India) using Next Generation Sequencing on an Illumina Platform and the sequencing end data was assembled with CLC Genomics Workbench v.6.0.5 with reads map back option. After that, the obtained sequencing results were analyzed for similarity search against nucleotide by BLASTN, NCBI program. Potential open reading frames (ORFs) were predicted using Genemark and Prokaryotic GeneMark.hmm version 3.25 respectively. The putative functions of those ORFs were analyzed by BLASTP, NCBI program. Using the BLASTP and PSI-BLAST programs against the non-redundant databases; the predicted ORFs were queried from translated sequences. The genome map of phage Sfk20 was drawn using the CG viewer server (http://cgview.ca/)^61^. The genomic comparison of phage Sfk20 with closely related myoviridae *Shigella* phages was illustrated in the form of a linear figure using Easyfig application (http://mjsull.github.io/Easyfig/files.html)^62^. In addition, tRNAs were predicted using ARAGORN (http://130.235.244.92/ARAGORN/)^63^ and tRNAscan-SE (http://lowelab.ucsc.edu/tRNAscan-SE/)^64^. For phylogenetic analysis two predicted ORFs were selected based on their amino acid sequences. We have chosen the amino acid sequence of baseplate wedge protein (ORF189, protein_id: QPP47184) and terminase large subunits (ORF163, protein_id: QPP47158). The amino acid sequences of Sfk20 were aligned with those of other reference Myoviridae bacteriophages using MUSCLE and then the phylogenetic tree was constructed using “ONE CLICK” at Phylogeny.fr.

### Nucleotide sequence accession number

The complete genome sequence of phage Sfk20 has been deposited in GenBank (Accession No. MW341595).

## Supplementary Information

Supplementary Information.
