# Supplementary material for: Morphological, biological, and genomic characterization of a newly isolated lytic phage Sfk20 infecting Shigella flexneri, Shigella sonnei, and Shigella dysenteriae1
Source: Sci Rep. 2021 Sep 29;11:19313. doi: 10.1038/s41598-021-98910-z (PMC8481304; doi:10.1038/s41598-021-98910-z)
Supplement: Supplementary file 1 — Supplementary Information. [file 41598_2021_98910_MOESM1_ESM.pdf]

Morphological, biological, and genomic characterization of a newly isolated lytic phage Sfk20 infecting *Shigella flexneri, Shigella sonnei,* and *Shigella dysenteriae1*

Bani Mallick[1], Payel Mondal[1] and Moumita Dutta[1]*

Supplementary Information

Supplementary Figure S1 | Scanning electron microscopic image of *Shigella flexneri* 2a infected with phage Sfk20 at different time points. (**A)** intact phage particles before adding to bacteria, (**B)** at early phase few phage particles attached to bacterial cell (white arrow indicates phage particles attached), (**C)** at mid phase many phage particles attached to bacterial cells, (**D)** at late phase bacterial cell disruption was observed.

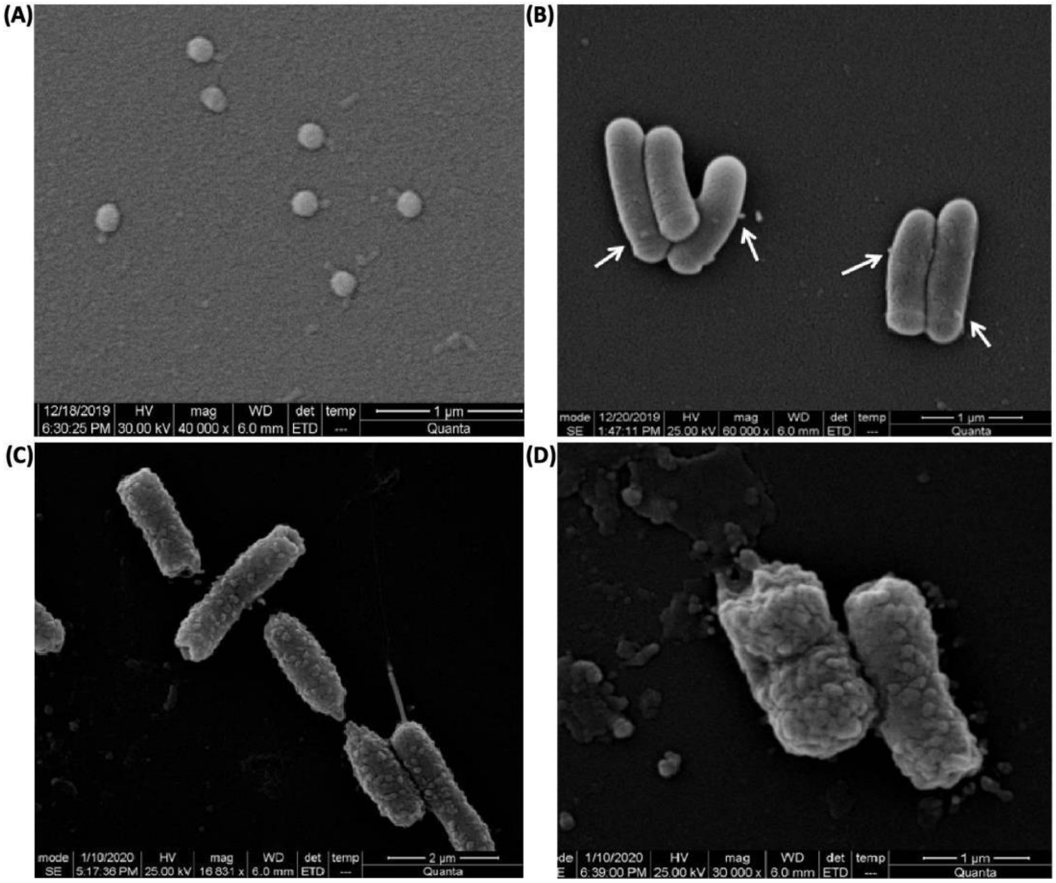

Supplementary Figure S2| Percentage of biofilm removal with phage Sfk20, antibiotic ampicillin and the combination of both with respect to untreated control. The values are shown as the mean ± SD of the values; Asterisks indicate significant reduction in biomass as measured by two-way ANOVA test (***P<0.001; **P<0.01; *P<0.05).

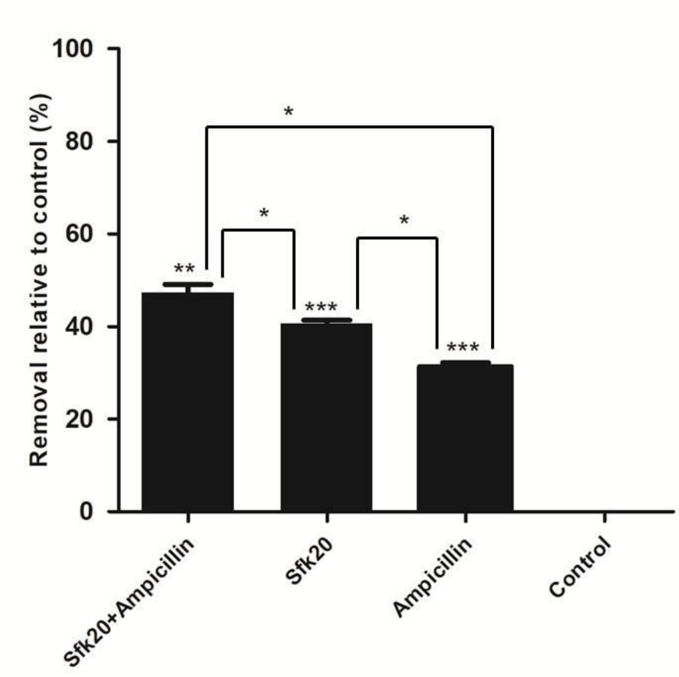

Supplementary Figure S3 | Protein profile of bacteriophage. Lane M, prestain protein ladder (abcam Prism Ultra Protein Ladder, ab116028), Lane S bacteriophage proteins.

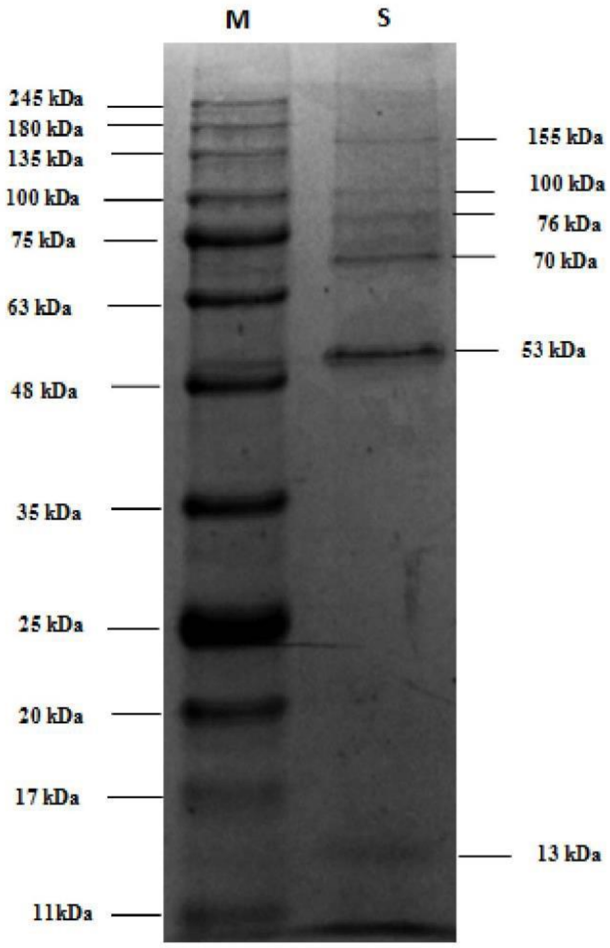

Supplementary Figure S4 | Phage genomic DNA was digested with different restriction enzymes. After overnight digestion the restriction fragments were separated by electrophoresis on 1% agarose gel stained with ethidium bromide. High range DNA ladder ranging from 250bp-25kb (Lane 1), uncut DNA (Lane 2), EcoRI(Lane 3), BamHI(Lane 4), HindIII(Lane 5),PstI(Lane 6), EcoRV(Lane 7), BglII(Lane 8) and MluI(Lane 9).

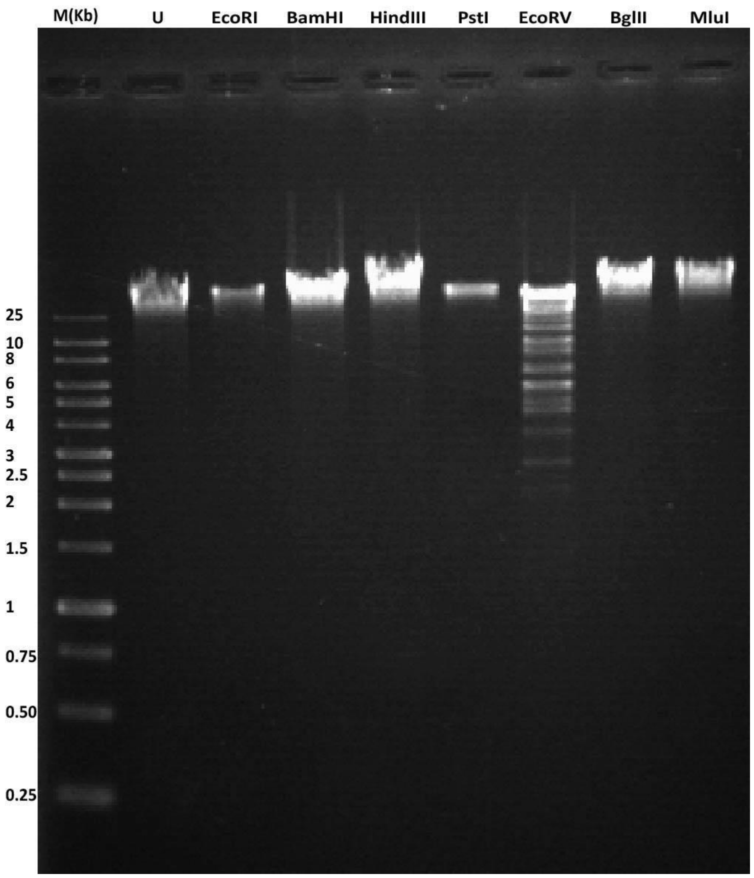

Supplementary Figure S5 | Phylogenetic trees of Sfk20 were constructed based on (**A)** baseplate wedge subunit and (**B)** the terminase large subunit(TerL) using "ONE CLICK" at Phylogeny.fr (https://www.phylogeny.fr)

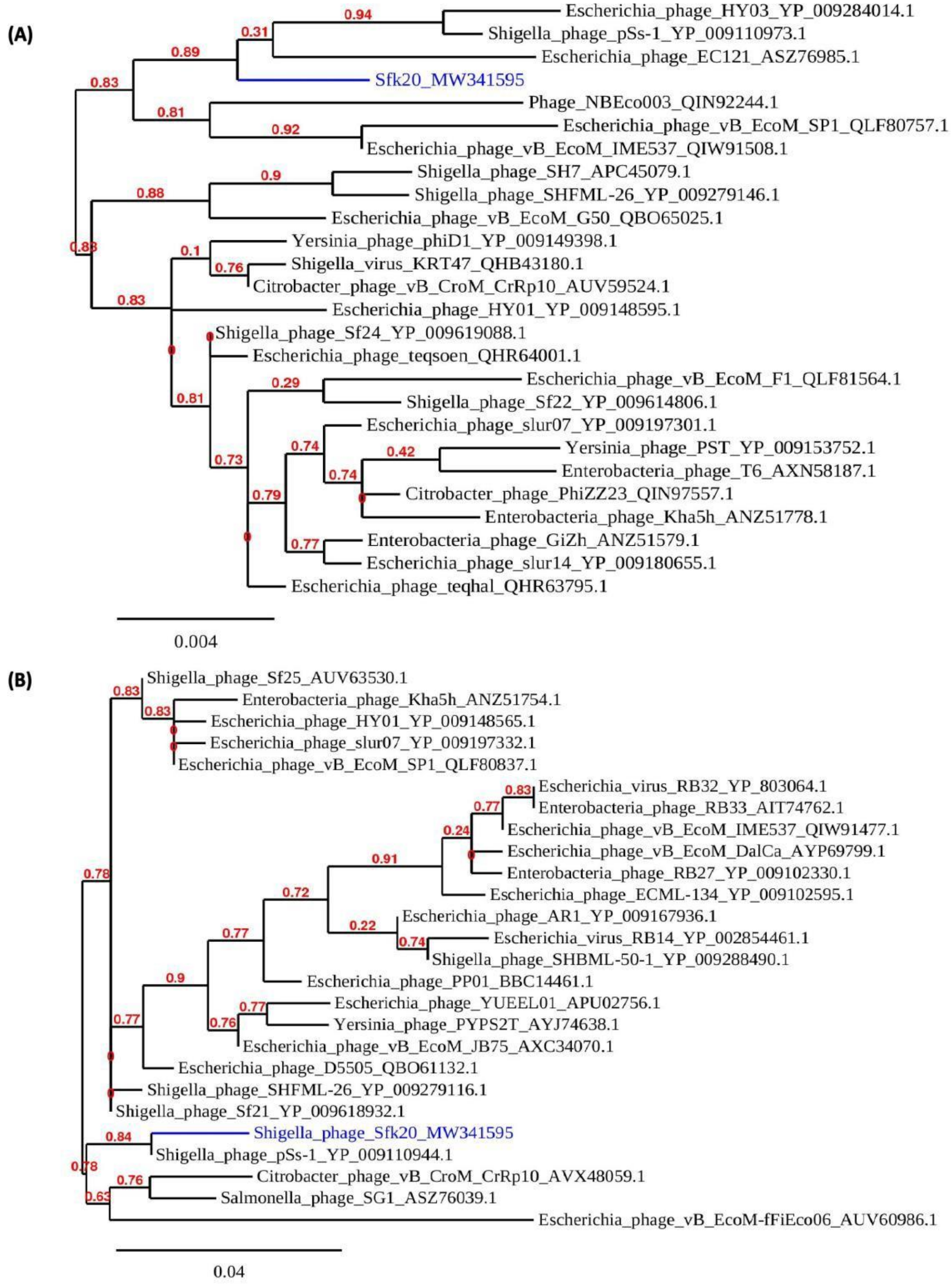

Table S1: All predicted ORFs, Their positions in Sfk20 phage genome, strand, their function and their role in phage life cycle.

ORFs	Start	Stop	Strand	Protein name	function in phage life cycle
ORF1	106	342	-	Hypothetical protein	
ORF2	342	923	-	dCMP deaminase	Nucleotide metabolism
ORF3	920	1255	-	Hypothetical protein	
ORF4	1255	1491	-	Hypothetical protein	
ORF5	1485	2012	-	Hypothetical protein	
ORF6	2075	2350	-	Hypothetical protein	
ORF7	2353	2553	-	Hypothetical protein	
ORF8	2546	2743	-	Hypothetical protein	
ORF9	2743	3648	-	3'-5' polynucleotide kinase	DNA damage repair
ORF10	3648	3968	-	Hypothetical protein	
ORF11	3972	4196	-	Hypothetical protein	
ORF12	4193	4492	-	Hypothetical protein	
ORF13	4489	4842	-	Hypothetical protein	
ORF14	4833	5336	-	Hypothetical protein	
ORF15	5401	6309	-	RNA ligase	RNA repair
ORF16	6578	6988	-	GIY-YIG nuclease family	Genome repair and recombination
ORF17	7016	8194	-	Aerobic NDP reductase small subunit	Genome replication and repair
ORF18	8246	10510	-	Ribonucleoside-diphosphate reductase subunit alpha	Genome replication
ORF19	10501	10788	-	Hypothetical protein	
ORF20	10781	11044	-	Hypothetical protein	
ORF21	11068	11928	-	Hypothetical protein	
ORF22	11928	12134	-	Hypothetical protein	
ORF23	12131	12712	-	Dihydrofolate reductase	Genome synthesis
ORF24	12712	12957	-	Hypothetical protein	
ORF25	12966	13301	-	Hypothetical protein	

ORF26	13312	13554	-	Hypothetical protein	
ORF27	13609	13974	-	Hypothetical protein	
ORF28	14018	14269	-	Hypothetical protein	
ORF29	14262	15197	-	Single stranded DNA-binding protein	Genome replication and recombination
ORF30	15297	15950	-	Hypothetical protein	
ORF31	15947	16285	-	Late promoter transcription accessory protein	Signal transduction and regulation
ORF32	16263	16532	-	Double-stranded DNA binding protein	Genome replication and recombination
ORF33	16541	17446	-	Hypothetical protein	
ORF34	22041	22559	+	Putative hinge connector of long tail fiber	Phage morphogenesis
ORF35	22622	23278	+	Hinge connector of long tail fiber distal connector	Phage morphogenesis
ORF36	23287	26598	+	Large distal long tail tube fiber subunit	Host infection
ORF37	26630	27409	+	Receptor recognition	Phage morphogenesis
ORF38	27440	28096	+	Holin	Host lysis
ORF39	28097	28369	-	Anti-sigma factor	Host transcription inhibition
ORF40	28382	28534	-	Hypothetical protein	
ORF41	28531	28809	-	Baseplate wedge tail fiber connector	Phage morphogenesis
ORF42	28799	28918	-	Hypothetical protein	
ORF43	29095	29391	-	Hypothetical protein	
ORF44	30189	30824	-	Hypothetical protein	
ORF45	30910	31059	-	Hypothetical protein	
ORF46	31056	32384	-	DNA gyrase subunit	Genome replication
ORF47	32854	32934	-	Activator of host PrrClysyl-tRNA endonuclease	RNA repair
ORF48	32934	33389	-	Nucleoid disruption protein	Host nucleoid destruction
ORF49	33449	33664	-	Hypothetical protein	
ORF50	33780	33977	-	Hypothetical protein	
ORF51	33985	34098	-	Hypothetical protein	
ORF52	34374	34580	-	Hypothetical protein	

ORF53	34663	35139	-	DNA endonuclease IV	Genome replication
ORF54	35153	35482	-	Hypothetical protein	
ORF55	35521	35715	-	Hypothetical protein	
ORF56	35744	36682	-	rIIB protector from prophage-induced early lysis	Genome replication
ORF57	36694	38871	-	hypothetical protein	
ORF58	38882	39085	-	Hypothetical protein	
ORF59	39140	40957	-	DNA topoisomerase II	DNA replication
ORF60	41027	41287	-	Hypothetical protein	
ORF61	41292	41663	-	Hypothetical protein	
ORF62	42264	42479	-	Hypothetical protein	
ORF63	42652	43140	-	Hypothetical protein	
ORF64	43217	43756	-	MotB	Transcription regulation
ORF65	43759	44259	-	Hypothetical protein	
ORF66	44323	45006	-	3'-5' exoribonuclease	Genome replication
ORF67	45006	45248	-	Hypothetical protein	
ORF68	45241	45486	-	Dextranase	Degradation of polysaccharide compounds
ORF69	45473	45733	-	Hypothetical protein	
ORF70	47056	47367	-	Hypothetical protein	
ORF71	47369	48115	-	Hypothetical protein	
ORF72	48233	48835	-	Putative adenylribosylating enzyme	Signal transduction and regulation
ORF73	48832	49455	-	ADP-ribosylase	Inhibit host transcription mechanism
ORF74	49523	49705	-	Hypothetical protein	
ORF75	49714	50184	-	molybdenum ABC transporter	Genome injection
ORF76	50177	50341	-	Hypothetical protein	
ORF77	50338	50493	-	Transcription modulator	Signal transduction and regulation
ORF78	50516	51001	-	Transcription modulator under heat shock	Signal transduction and regulation
ORF79	50982	51191	-	Hypothetical protein	

ORF80	51348	51560	-	Hypothetical protein	
ORF81	51659	51904	-	Head decoration	Phage morphogenesis
ORF82	51977	52495	-	Hypothetical protein	
ORF83	52567	52767	+	Hypothetical protein	
ORF84	52764	53792	-	DNA primase	Genome replication
ORF85	53795	53959	-	Hypothetical protein	
ORF86	53972	54265	-	Spackle periplasmic protein	Host lysis
ORF87	54324	54581	-	Hypothetical protein	
ORF88	54583	54765	-	hypothetical protein	
ORF89	54824	56251	-	DNA primase/helicase	Genome replication
ORF90	56261	56605	-	Head vertex assembly chaperone	Phage morphogenesis
ORF91	56598	57779	-	Putative RecA-like recombination protein	Genome repair
ORF92	57857	58699	-	Beta -glucosyl-HMC-alpha-glucosyl-transferase	Genome modification
ORF93	58696	59436	-	dCMPhydroxymethylase	Genome replication
ORF94	59590	59841	-	superinfection immunity protein	Superinfection exclusion
ORF95	59849	60229	-	hypothetical protein	
ORF96	60412	63108	-	DNA polymerase	Genome replication
ORF97	63187	63555	-	Translational repressor	Genome replication
ORF98	63557	64120	-	Clamp loader small subunit	Genome replication
ORF99	64122	65081	-	Replication factor C small subunit	Genome replication
ORF100	65131	65817	-	Sliding clamp DNA polymerase accessory protein	Genome replication
ORF101	65873	66262	-	RNA polymerase binding protein	Genome replication
ORF102	66272	66460	-	Hypothetical protein	
ORF103	66516	68066	-	Hypothetical protein	
ORF104	68195	68401	-	Hypothetical protein	
ORF105	68382	68645	-	Hypothetical protein	
ORF106	68642	69661	-	Hypothetical protein	
ORF107	69838	71040	-	alpha glucosyl transferase	Genome modification
ORF108	71456	71773	-	Hypothetical protein	

ORF109	71775	71993	-	Hypothetical protein	
ORF110	71977	72534	-	Sigma factor	Signal trunsduction and regulation
ORF111	72613	72882	-	Hypothetical protein	
ORF112	72879	73094	-	Hypothetical protein	
ORF113	73097	73423	-	Hypothetical protein	
ORF114	73476	73676	-	Hypothetical protein	
ORF115	73677	73808	-	Hypothetical protein	
ORF116	73816	74109	-	Hypothetical protein	
ORF117	74102	74278	-	Hypothetical protein	
ORF118	74436	74744	-	Glutaredoxin	Reducing agent for phage induced riboucleotide reductase
ORF119	74747	74959	-	Hypothetical protein	
ORF120	75075	75545	-	Anaerobic nucleotide reductase subunit	Genome replication
ORF121	75542	77359	-	Ribonucleotide reductase of class III large subunit	Genome replication
ORF122	77356	77829	-	Endonuclease	Genome replication
ORF123	77872	78048	-	Hypothetical protein	
ORF124	78048	78494	-	Protease inhibitor	Inhibit host protease
ORF125	78618	78938	-	Hypothetical protein	
ORF126	78949	79119	-	Hypothetical protein	
ORF127	79122	79337	-	Hypothetical protein	
ORF128	79334	79597	-	Thioredoxin	Signal transduction and modification
ORF129	79599	79841	-	Hypothetical protein	
ORF130	79828	80145	-	Hypothetical protein	
ORF131	80142	81071	-	Hypothetical protein	
ORF132	81124	82125	-	Hypothetical protein	
ORF133	83214	84104	-	Hypothetica l protein	
ORF134	84112	84513	-	Thioredoxin	Signal transduction and modification
ORF135	84569	85096	-	Hypothetical protein	

ORF136	85157	85459	-	Hypothetical protein	
ORF137	85561	86529	-	Hypothetical protein	
ORF138	86595	87605	-	Hypothetical protein	
ORF139	87605	88066	-	Hypothetical protein	
ORF140	88069	88590	-	Hypothetical protein	
ORF141	88597	89130	-	Hypothetical protein	
ORF142	89157	89261	-	Molybdopterin -guanine dinucleotide biosynthesis	
ORF143	89322	89495	-	Hypothetical protein	
ORF144	89485	89679	-	Hypothetical protein	
ORF145	89682	89885	-	Hypothetical protein	
ORF146	89885	90073	-	Hypothetical protein	
ORF147	90169	90555	-	Hypothetical protein	
ORF148	90552	90845	-	rI lysis inhibition	Host lysis
ORF149	90858	91070	-	Hypothetical protein	
ORF150	91113	91694	-	Thymidine kinase	Genome replication
ORF151	91696	91884	-	Hypothetical protein	
ORF152	91881	92066	-	Hypothetical protein	
ORF153	92063	92236	-	Hypothetical protein	
ORF154	92436	92648	-	Hypothetical protein	
ORF155	92620	93099	-	Hypothetica l protein	
ORF156	93436	93981	-	transglycosylase SLT domain-containing protein	Host cell lysis
ORF157	93989	94450	-	Site -specific Rnase	Host mRNA degradation
ORF158	94510	94788	-	Hypothetical protein	
ORF159	94788	95054	-	Hypothetical protein	
ORF160	95047	95268	-	Hypothetical protein	
ORF161	95268	95630	-	Hypothetical protein	
ORF162	95637	95966	-	Hypothetical protein	
ORF163	95963	96532	-	large terminase subunit	Genome packaging
ORF164	96656	97129	-	Hypothetical protein	
ORF165	97139	97555	-	Pyrimidine dimer DNA glycosylase	

				endonuclease V	
ORF166	97615	98109	-	Endolysin/lysozyme	Host lysis
ORF167	98146	98586	-	nudix hydrolase	Hydrolase activity
ORF168	98583	99071	-	Putative transmembrane region domain containing protein	Phage morphogenesis
ORF169	99068	99430	-	Hypothetical protein	
ORF170	99412	99804	-	Hypothetical protein	
ORF171	99773	100393	-	Hypothetical protein	
ORF172	100435	101028	-	Hypothetical protein	
ORF173	101086	101421	-	Hypothetical protein	
ORF174	101477	101740	-	Hypothetical protein	
ORF175	101981	102544	-	Hypothetical protein	
ORF176	102671	103144	-	Hypothetical protein	
ORF177	103529	103879	-	Hypothetical protein	
ORF178	104810	105325	-	Hypothetical protein	
ORF179	105328	105618	-	Hypothetical protein	
ORF180	105621	106001	-	Hypothetical protein	
ORF181	106003	106188	-	Hypothetical protein	
ORF182	106259	106516	-	Hypothetical protein	
ORF183	107044	107286	-	Hypothetical protein	
ORF184	107286	108011	-	Deoxynucleoside monophosphate kinase	Genome replication
ORF185	108698	109522	-	DNA end protector	Genome replication and modification
ORF186	109522	109974	-	Head completion	Phage morphogenesis
ORF187	110022	110612	+	Baseplate wedge subunit	Phage morphogenesis
ORF188	113794	115137	+	Baseplate wedge	Phage morphogenesis
ORF189	115134	118232	+	Baseplate wedge subunit	Phage morphogenesis
ORF190	118723	119229	+	Hypothetical protein	
ORF191	119293	120159	+	Baseplate wedge completion tail fiber socket	Phage morphogenesis
ORF192	120159	121964	+	Hypothetical protein	
ORF193	122620	124170	+	Hypothetical protein	

ORF194	124180	125637	+	Hypothetical protein	
ORF195	125670	126599	+	Neck protein	Phage morphogenesis
ORF196	126601	127371	+	Hypothetical protein	
ORF197	127413	128231	+	Tail sheath stabilizer and completion protein	Phage morphogenesis
ORF198	128240	128734	+	Small terminase protein	Genome packaging
ORF199	128718	130550	+	Putative terminase subunit	Genome packaging
ORF200	130582	132561	+	Tail sheath	Phage morphogenesis
ORF201	132678	133169	+	Putative tail tube monomer	Phage morphogenesis
ORF202	133253	134827	+	Hypothetical protein	
ORF203	134827	135084	+	Prohead core	Phage morphogenesis
ORF204	135084	135509	+	Capsid and scaffold protein	Virion assembly
ORF205	135509	136147	+	Prohead core scaffolding protein and protease	Phage morphogenesis
ORF206	136178	136987	+	Prohead assembly	Phage morphogenesis
ORF207	137006	138571	+	Major capsid protein	Phage morphogenesis
ORF208	138655	139938	+	Hypothetical protein	
ORF209	139968	140972	-	RNA ligase 2	Genome replication
ORF210	140982	141260	-	Hypothetical protein	
ORF211	141247	141462	-	Hypothetical protein	
ORF212	141552	143546	-	PKD domain protein	Phage morphogenesis
ORF213	143556	144236	-	Hypothetica l protein	
ORF214	144287	145798	+	Hypothetical protein	
ORF215	145824	146054	+	ATP-dependent DNA helicase	Genome replication
ORF216	146306	146530	-	Hypothetical protein	
ORF217	146530	146943	-	Recombination mediator protein	Genome modification
ORF218	147010	147408	-	Hypothetical protein	
ORF219	147408	148034	-	Baseplate hub subunit	Phage morphogenesis
ORF220	148085	148834	+	Baseplate protein	Phage morphogenesis
ORF221	148834	150009	+	Hypothetical protein	
ORF222	150029	150487	+	Baseplate distal hub subunit	Phage morphogenesis
ORF223	150484	152256	+	Hypothetical protein	

ORF224	152757	153359	+	Hypothetical protein	
ORF225	153359	154324	+	Hypothetical protein	
ORF226	154353	154643	-	Hypothetical protein	
ORF227	154704	156761	-	Hypothetical protein	
ORF228	156765	158858	-	ADP-ribosyltransferaseexoenzyme	Genome replication
ORF229	158911	159099	-	Hypothetical protein	
ORF230	159096	160556	-	DNA ligase	Genome replication
ORF231	160553	160822	-	Hypothetical protein	
ORF232	160822	161661	-	Hypothetical protein	
ORF233	161658	162116	-	Hypothetical protein	
ORF234	162109	162324	-	Hypothetical protein	
ORF235	162329	162616	-	Hypothetical protein	
ORF236	162658	163023	-	Hypothetical protein	
ORF237	163091	163423	-	Hypothetical protein	
ORF238	163534	163710	-	Hypothetical protein	
ORF239	163905	164153	-	Hypothetical protein	
ORF240	164301	164636	-	Co-chaperone GroES	Signal transduction and regulation
ORF241	164693	164863	-	Hypothetical protein	

Table S2: Comparison of genomic properties of phage Sfk20 with closely related Myoviridae phages

**Phage name**	**Sfk20 (present study)**	**pSs-1**	**SH7**	**SfPhi 01**	**Sf21**	**Sf23**
Genome length (bp)	164878	164999	164870	168000	166002	167678
GC content (%)	35.6	35.5	35.4	35.4	35.5	35.4
Host species	*S. flexneri 2a*	*S. sonnei*	*S. flexneri*	*S. flexneri*	*S. flexneri*	*S. flexneri*
Identity (%)	-	97.91	96.03	95.34	95.40	95.46
Accession Number	MW341595	NC_025829	KX828711	LC465543	NC_042077	MF158046

Table S3: Comparison of some recently published *Shigella* phages based on biological properties

**Phage name**	**Genome length (bp)**	**GC content (%)**	**Total/identified ORF**	**t-RNA**	**Latent period (min)**	**Burst size(PFU/ cell)**	**Host sensitivity towards *Shigella* strains**
**Sfk20 (present study)**	164878	35.6	241/92	10	20	123	*S. flexneri, S. sonnei, S.dysenteriae*
**pSs-1**	164999	35.5	266/121	10	25	97	*S. flexneri, S. sonnei*
**pSf-1**	51,821	44.0	94/26	-	10	86.86	*S. flexneri, S. sonnei, S. boydii*
**pSf-2**	50,109	45.4	83/22	-	30	16	*S. flexneri*
**Sfin-1**	50,403	45.2	82/23	-	5	27-28	*S. flexneri, S. sonnei, S.dysenteriae*
**vB_SflM _004**	85,887	38.6	135/48	-	30	139	*S. flexneri, S. sonnei*
